# Risk Taking by Adolescents with Attention-Deficit/Hyperactivity Disorder (ADHD): a Behavioral and Psychophysiological Investigation of Peer Influence

**DOI:** 10.1007/s10802-020-00666-z

**Published:** 2020-06-30

**Authors:** Tycho J. Dekkers, Arne Popma, Edmund J.S. Sonuga-Barke, Helena Oldenhof, Anika Bexkens, Brenda R. J. Jansen, Hilde M. Huizenga

**Affiliations:** 1 Department of Psychology, University of Amsterdam, Nieuwe Achtergracht 129B, 1018WS, Amsterdam, The Netherlands; 2 Department of Forensic Psychiatry and Complex Behavioral Disorders, De Bascule, Academic Center for Child- and Adolescent Psychiatry, Amsterdam, The Netherlands; 3 Amsterdam UMC, Department of Child- and Adolescent Psychiatry, Free University Medical Center (VUmc), Amsterdam, The Netherlands; 4 Department of Child & Adolescent Psychiatry, Institute of Psychiatry, Psychology & Neuroscience, King’s College, London, UK; 5 Department of Developmental and Educational Psychology, Leiden University, Leiden, The Netherlands; 6 Department of Child and Adolescent Psychiatry, GGZ Delfland, Center for Psychiatry, Delft, The Netherlands; 7 Amsterdam Brain and Cognition Center, University of Amsterdam, Amsterdam, The Netherlands; 8 Research Priority Area Yield, University of Amsterdam, Amsterdam, The Netherlands

**Keywords:** Attention-deficit/hyperactivity disorder (ADHD), Risk taking, Peer influence, Autonomic reactivity, Stress, Balloon analogue risk task (BART)

## Abstract

Adolescents with ADHD demonstrate increased risk-taking behavior (RTB) like substance abuse and dangerous traffic conduct. RTB in adolescence is more likely under peer influence. The current investigation (1) tests the hypothesis that adolescents with ADHD are particularly susceptible to such influence and (2) tests whether groups differed in autonomic reactivity to peer influence. Adolescent boys between 12 and 19 years with (*n* = 81) and without (*n* = 99) ADHD performed the Balloon Analogue Risk Task twice. In the peer condition, a highly credible virtual peer manipulation that encouraged risk taking was added, in the solo condition this was absent. Autonomic reactivity was indexed by heart rate (HR), pre-ejection period (PEP) and respiratory sinus arrhythmia (RSA). *All* adolescents engaged in more risk taking in the peer condition relative to solo condition. Autonomic differences between groups were only found on PEP: a stronger sympathetic response to peer influence was observed in typically developing adolescents relative to adolescents with ADHD. Increased physiological stress (as indexed by PEP) in the peer relative to the solo condition predicted peer-induced risk taking in all adolescents. We conclude that susceptibility to peer influence is not exaggerated in ADHD but rather reflects a general tendency of adolescents. As adolescents experiencing peer influence as stressful are most susceptible to peer influence, we suggest that increasing resistance to peer influence may be an important treatment aim for these adolescents specifically.

**Electronic supplementary material:**

The online version of this article (10.1007/s10802-020-00666-z) contains supplementary material, which is available to authorized users.

## Introduction

Attention-Deficit/Hyperactivity Disorder (ADHD) is a neurodevelopmental disorder, defined by persistent patterns of inattention and/or hyperactivity/impulsivity, causing impairment in several life domains (American Psychiatric Association, 2013). ADHD is one of the most prevalent psychiatric disorders in adolescence (Polanczyk et al., 2014), and besides personal impairment, the financial burden of ADHD on society is high (Robb et al., 2011). A substantial part of these costs is related to risk-taking behavior (RTB; Matza et al., 2005). ADHD is associated with several forms of real life risk-taking behavior (RTB) like substance abuse, reckless driving, gambling or unsafe sex (see Pollak et al., 2019 for a review). Also on laboratory risk-taking tasks, children and adolescents with ADHD demonstrate more risk taking than controls (see Dekkers et al., 2016 for a meta-analysis). Given the vulnerability of adolescents to RTB in general (Crone & Dahl, 2012), and considering that adolescents with ADHD seem even more vulnerable to RTB, it is important to study underlying mechanisms that can help understand and ultimately reduce RTB in this group. In the current investigation, we focus on one such putative mechanism – peer influence.

### Susceptibility to Peer Influence

Adolescence is a period of elevated peer influence on RTB (Somerville, 2013; Steinberg & Morris, 2001). Several experimental studies demonstrate that the presence of and/or the encouragement by peers increases risk taking in typically developing adolescents (Cavalca et al., 2013; Chein et al., 2011; Gardner & Steinberg, 2005; Rhodes et al., 2015; Smith et al., 2014; van Hoorn et al., 2017; Weigard et al., 2014). Real-life data reveal that among adolescent drivers, risky driving and the risk of fatal injuries increases with more same-aged passengers in the car (Chen et al., 2000; Ouimet et al., 2010; Simons-Morton et al., 2011, 2012).

According to the widely used dual-systems model, this increase in RTB during adolescence can be explained by a more rapid development of socioemotional brain systems relative to cognitive control systems, causing an increase in reward-seeking behavior (Steinberg, 2010; Strang et al., 2013). Because peers trigger socioemotional brain systems by activating reward-related regions like the ventral striatum (Chein et al., 2011; Gardner & Steinberg, 2005; Somerville, 2013), and peer presence is associated with an increase in the subjective value of immediate rewards (Albert et al., 2013), more cognitive control is required to control behavior in the presence of peers. As ADHD is characterized by pronounced inhibitory deficits and a delay in cortical maturation (Barkley, 1997; Lijffijt et al., 2005; Rubia et al., 2005; Shaw et al., 2007, 2018), it follows that a larger imbalance between these brain systems is to be expected in this group (Sonuga-Barke, 2003), potentially making adolescents with ADHD unusually susceptible to peer influence.

Social factors may also be related to elevated susceptibility to peer influence in adolescents with ADHD. Generally, adolescents with social problems and weak social skills are most susceptible to peer influence (Allen et al., 2012; Steinberg et al., 1994; Urberg et al., 2003). Adolescents with ADHD experience myriad social problems. Specifically, ADHD is associated with a wide range of socially inadequate behaviors such as social intrusiveness, difficulties attuning social behavior, violation of social rules, socially dominant behavior, verbal aggression, talking when inappropriate, and being easily distracted in conversation (Huang-Pollock et al., 2009; Nijmeijer et al., 2008). These behaviors are associated with lower popularity among peers (Bagwell et al., 2001; Hoza et al., 2005). Children with ADHD also encounter more peer rejection relative to their peers without ADHD (de Boo & Prins, 2007; Hoza, 2007), which persists into adolescence (Bagwell et al., 2001). Peer rejection in turn may increase susceptibility to peer influence by a process called reputation management: displaying RTB to gain status and/or to avoid rejection (Brechwald & Prinstein, 2011). Indeed, peer rejection in adolescents with ADHD is associated with externalizing disorders, antisocial behavior and substance use (Greene, 1997; Mikami & Hinshaw, 2006; Mrug et al., 2012).

To summarize, several lines of evidence lead us to hypothesize that adolescents with ADHD are more susceptible to peer influence, which has the potential to increase RTB. This hypothesis has – to our knowledge – never been tested. In the current preregistered study, we therefore investigate whether adolescents with ADHD are more susceptible to peer influence than are typically developing (TD) adolescents. To do this, we developed a paradigm combining risk taking and peer influence. A virtual risk-encouraging peer was integrated in the Balloon Analogue Risk Task (Lejuez et al., 2003), which was administered twice (peer and solo condition).

In adolescence, peer influence typically imposes stress (Byrne et al., 2007), potentially because of the fear of exclusion and feelings of need-to-belong that peers may trigger (Baumeister et al., 2005; Pickett et al., 2004). For example, in typically developing adolescents, unexpected social rejection is associated with a parasympathetic response (Gunther Moor et al., 2014). In adolescents with ADHD, the physiological effects of peer influence are yet unknown. As adolescents with ADHD experience more peer rejection relative to TD adolescents (Bagwell et al., 2001; de Boo & Prins, 2007), we reasoned that peer influence may elicit increased stress (i.e., ANS reactivity) in the ADHD group. However, a recent meta-analysis indicates that ADHD is mostly associated with physiological *hypo*activation, although results are mixed and most studies investigated this during resting state or cognitive tasks; evidence on physiological reactivity to social information is heterogeneous and inconclusive (Bellato et al., 2020).

In the non-preregistered part of current study, we assess physiological responding to peer influence by measuring autonomic nervous system (ANS) reactivity. We tested if adolescents with and without ADHD differed in ANS reactivity to peer influence. In addition, as fear of exclusion can promote risk taking (Pickett et al., 2004), we reasoned that increased ANS reactivity is linked to more risk taking in the peer condition, thereby testing the link between physiological and behavioral effects of peer influence.

## Method

The behavioral part of the study was preregistered via AsPredicted (Supplementary Materials 1 and https://aspredicted.org/gh3u4.pdf).

### Participants

Participants were 180 adolescent boys,^1^ ages 12–19, with (*N* = 81) and without (*N* = 99) ADHD (see Table 1 for group characteristics). Adolescents were excluded if their estimated IQ was below 80. For the ADHD group, adolescents were included if they (a) had been diagnosed with ADHD before by a mental healthcare professional, as indicated by their parents/caretakers; (b) scored outside the normal range on the inattention or hyperactivity/impulsivity subscale of the Disruptive Behavior Disorders Rating Scale (DBDRS) (Oosterlaan et al., 2000), administered to one of the parents/caretakers *and* (c) scored above the diagnostic threshold for any ADHD presentation according to the parent Diagnostic Interview Schedule for Children (DISC-IV) (Shaffer et al., 2000). Adolescents were requested to refrain from stimulant medication for 24 (if using Methylphenidate) or 48 (if using dextroamphetamine) hours, to reach complete wash-out (Greenhill & Ford, 1998; Wong & Stevens, 2012). Adolescents using atomoxetine, clonidine and anti-psychotic medication were excluded.

**Table 1:** Group characteristics

	ADHD (*n* = 81)	TD (*n* = 99)	
Age	15.0 (1.8)	15.1 (1.4)	*t*(148^a^) = 0.54, *n.s.*
IQ	103.4 (13.7)	101.8 (12.9)	*t*(178) = −0.79, *n.s.*
SES	5.7 (.8)	5.7 (.8)	*t*(178) = 0.12, *n.s.*
DBDRS inattention	16.1 (1.4)	10.9 (1.2)	*t*(178) = −26.4***
DBDRS hyperactivity/impulsivity	15.2 (1.9)	10.6 (1.1)	*t*(127^a^) = −19.6***
DBDRS ODD	13.6 (2.2)	10.8 (1.2)	*t*(119^a^) = −9.7***
DBDRS CD	13.7 (2.7)	11.3 (1.2)	*t*(108^a^) = −7.4***
DISC ADHD presentation (C/I/HI)	40/39/2	–	–
Medication (Y/N)	58/23	–	–
DISC disruptive behavioral disorders	31%	–	–
DISC substance use disorder	3%	–	–
DISC anxiety disorder	30%	–	–
DISC mood disorder	6%	–	–
DISC tic disorder	16%	–	–
DISC enuresis/encopresis	1%	–	–
DISC eating disorder	1%	–	–
Autistic symptoms	10.4 (6.0)	1.9 (2.4)	*t*(100^a^) = −12.0***
Smoking, cigarettes/day	0.6 (2.9)	0.6 (2.7)	*t*(178) = −0.15, *n.s.*
Exercise, hours/week	5.4 (5.1)	5.7 (2.9)	*t*(121) = 0.47, *n.s.*
Body mass index (BMI)	19.2 (2.9)	20.1 (2.9)	*t*(176^b^) = 2.0*

^a^The assumption of equal variances was violated, therefore DF differs

^b^BMI information was unavailable for two participants

^#^*p* < 0.10; *p < 0.05; **p < 0.01; ***p < 0.001

Abbreviations: *ADHD* Attention-Deficit/Hyperactivity Disorder, *C* Combined, *CD* Conduct Disorder, *DBD* Disruptive Behavior Disorder (i.e., ODD and/or CD), *DBDRS* Disruptive Behavior Disorders Rating Scale, *DISC* Diagnostic Interview Schedule for Children, *HI* Hyperactive/Impulsive, *I* Inattentive, *ODD* Oppositional Defiant Disorder, *SES* socio-economic status, *TD* Typically Developing control group. Standard deviations in parentheses.

**Fig. 1:**
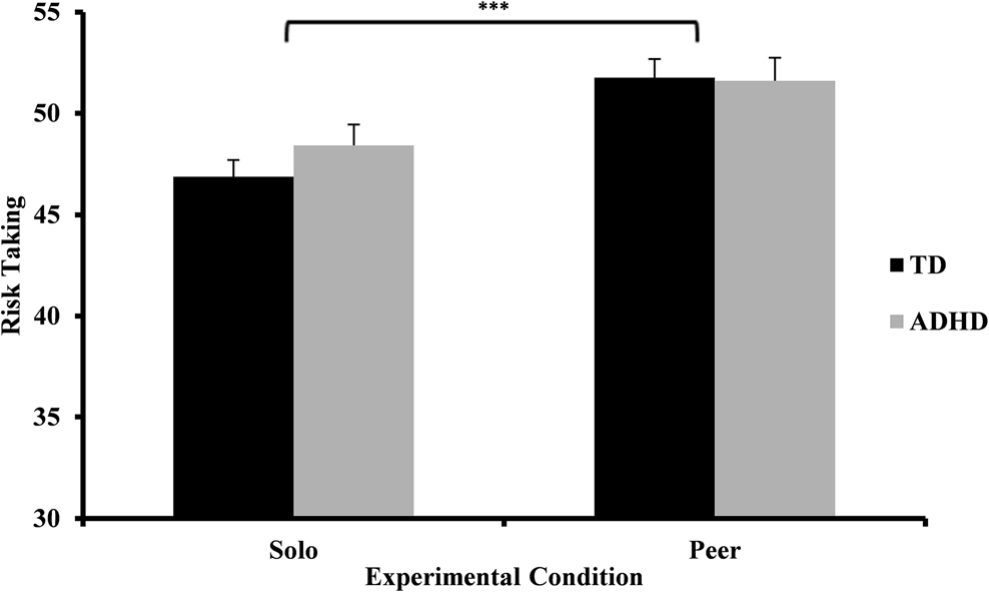
Risk taking (y-axis) across experimental conditions (x-axis) in adolescents with ADHD (grey bars) and typically developing (TD; black bars) adolescents

For the TD group, adolescents were excluded if they (a) had a lifetime diagnosis of ADHD, Oppositional Defiant Disorder (ODD) or Conduct Disorder (CD) *or* (b) scored (sub)clinical on any DBDRS subscale (inattention, hyperactivity/impulsivity, oppositional behavior, conduct problems). All adolescents and their legal caretakers gave informed consent and the study was approved by the Institutional Review Board of the University of Amsterdam (department of developmental psychology).

### Materials

#### Intelligence

A short version of the Dutch Wechsler Intelligence Scale for Children-III (WISC-III-NL, subtests Block Design and Vocabulary) (Kort et al., 2002; Wechsler, 1991) was administered to adolescents up to 16 years. The short version of the Dutch Wechsler Adult Intelligence Scale-IV (WAIS-IV, subtests Vocabulary and Matrix Reasoning) (Wechsler, 2008) was used with adolescents from 16 years. Reliability of both short versions is adequate, and correlations with full-scale IQ are high (Pierson et al., 2012; Sattler, 2001).

#### Socio-Economic Status (SES)

SES was established based upon the level of education of both parents, using Verhage’s seven-point classification (Verhage, 1964).

#### Disruptive Behavior Disorder Rating Scale (DBDRS)

As a screener for ADHD, ODD and CD symptoms, the Dutch version of the DBDRS (Oosterlaan et al., 2000; Pelham et al., 1992) was completed by one of the parents/caretakers. The DBDRS yields scores on subscales inattention, hyperactivity/impulsivity, ODD and CD, which were classified as normal (percentiles <90), subclinical (percentiles 90–94) or clinical (percentiles >94). Psychometric properties are adequate as indicated by high internal consistency of the subscales (*α*’s between 0.75 and 0.96) and a factor structure highly similar to the DSM (Oosterlaan et al., 2000; Pelham et al., 1992).

#### Diagnostic Interview Schedule for Children (DISC-IV)

As assessment of ADHD and comorbidity, the Dutch version of the DISC-IV (Ferdinand & van der Ende, 1998; Shaffer et al., 2000), sections anxiety disorders, mood disorders, schizophrenia, disruptive behavior disorders, substance use disorders and miscellaneous disorders, was administered to one of the parents/caretakers. This semi-structured interview assesses DSM-IV symptomatology, and has a good test-retest reliability (*κ* = 0.79) and was predictive of clinicians’ ratings (*κ* = 0.72) (Shaffer et al., 2000).

#### Autistic Symptoms

To screen for autistic symptoms, and to potentially specify whether peer effects are attributable to ADHD or only to comorbid autistic symptoms, the autism subscale of the Social Emotional Questionnaire (in Dutch, SEV; Scholte & van der Ploeg, 2007) was used, which consists of ten items and was completed by one of the parents/caretakers. Reliability, criterion validity and construct validity were qualified as ‘good’ (Scholte & van der Ploeg, 2007).

#### Balloon Analogue Risk Task

The Balloon Analogue Risk Task (BART; Lejuez et al., 2003) was used to measure risk taking. In this task, adolescents pump a balloon on the screen. With every pump, the balloon grew and its monetary value increasde by €0,01; however, the probability of exploding also increased with every pump. If the balloon exploded, the adolescent earned nothing and continued to the next balloon. Adolescents could “cash” the value of the balloon at any time; the total amount of cashed money was paid out in cash directly after the last session (pay-out per session was €8,- on average). The BART consisted of 30 trials, and the array of explosion points was determined randomly with explosion points ranging between 5 and 128 (*M* = 64). The same array was used for every adolescent to enhance comparisons. The mean number of pumps on non-exploded balloons was used as index of risk taking (Lejuez et al., 2003). In young adults, BART performance correlated with self-reported real-life risk-taking behaviors, such as smoking, drug and alcohol use and gambling, with correlations ranging from *r* = 0.28 to *r* = 0.44 (Lejuez et al., 2002) and in adolescents, BART performance predicted alcohol use 2 years later (MacPherson et al., 2010).

#### Peer Influence Manipulation

A peer influence component was added to the BART. Adolescents were told that a peer who was the same age and sex in another location tried to predict the performance of the participant, without actually meeting each other. There was a short, standardized introduction over WhatsApp on a smartphone that was provided to the adolescent, and the adolescent was told that the peer was watching him via a camera positioned behind him. After the practice block (3 trials without cashing, 3 trials with cashing) and halfway of the task (i.e., after 15 trials), the adolescent was again allowed to communicate with the peer; at both occasions the peer send a risk-encouraging message (i.e., “I would pump the balloon a little more, for me that worked well”). In fact, the peer was a confederate of the study. This manipulation was based on previous studies that successfully used digital peer manipulations (Smith et al., 2014; Weigard et al., 2014). Comprehensive piloting of the protocol ensured that the manipulation was very credible, which was checked by coding of the content of all individual WhatsApp conversations (protocol and details on coding of the content of conversations can be found in Supplementary Materials 2). Participants and their parents were debriefed after completion of the study.

#### Physiological Measures

Autonomic activity during baseline and BART was measured with VU-AMS (Vrije Universiteit Ambulatory Monitoring System; de Geus et al., 1995). Based on ECG (electrocardiogram) and ICG (impedance cardiogram) registration, three indices were derived. The heart rate (beats per minute) was determined based on R-peak time series from the ECG. Heart rate variability was indexed by respiratory sinus arrhythmia (RSA): the difference between the longest inter-beat interval during expiration and the shortest during inspiration (Thayer et al., 2012). The pre-ejection period (PEP) was defined as the interval between the start of left ventricular depolarization (q-wave onset in the ECG) and opening of the aortic valve (b-point in the ICG) (Oldenhof et al., 2018; Van Lien et al., 2013). HR is a measure of general autonomic activity, and results from the interaction between the PNS and SNS. PEP is a measure of sympathetic nervous system (SNS) activity specifically, with shorter PEP values indicating higher SNS activity. RSA is a measure of parasympathetic nervous system (PNS) activity specifically, with smaller RSA values indicating lower PNS activity.

VU-DAMS (v4.0) algorithms were used for ECG/ICG scoring. All data were checked manually for ECG-abnormalities: missing R-peaks were added, premature ventricular contractions and premature atrial contractions were removed and B-point identification was checked for each averaged ICG complex (Oldenhof et al., 2018). RSA was specified as missing if >50% of the respiration signal was identified as irregular by the algorithm. VU-DAMS automatically registered RSA as 0 when no difference between the shortest and the longest inter-beat interval could be detected during a respiration cycle. PEP was specified as missing if >50% of the beats within an averaged ICG complex were discarded because of poor signal quality. Scoring was performed by trained researchers and assistants. In case of doubt, data were scored using group-consensus.

Baseline values of autonomic activity were obtained in both sessions (i.e., BART with and without peer influence manipulation) during presentation of a 5-min film clip showing peaceful aquatic sceneries, accompanied with classical music (Coral Sea Dreaming, Small World Music Inc.), which was successfully used before to assess baseline autonomic activity (Piferi et al., 2000). The percentage of change from baseline to task (((task – baseline)/baseline) × 100%) was used as outcome measure for reactivity of HR, PEP and RSA (indicated by ΔHR, ΔPEP, and ΔRSA, respectively).

### Procedure

Adolescents were recruited via schools and mental healthcare institutions. After providing consent, one of the parents/caretakers completed the DBDRS and SEV online. Adolescents were tested during three sessions in a quiet room, either at school, the mental healthcare institution or the university. In the first session (1 h), adolescents underwent intelligence testing, completed questionnaires and were weighed and measured. The second and third session (3 h each) were highly similar to each other. The main difference was that in one session the BART was administered without a virtual peer (i.e., solo session) whereas in the other session the peer influence manipulation was added (i.e., peer session). The order of tasks (solo, peer) was counterbalanced. In both sessions, VU-AMS measures were attached first, followed by a 25-min resting period during which the adolescent completed questionnaires (different questionnaires in different sessions) and read magazines. During these 25 min, there was no communication between the adolescent and the experimenter. Then, the adolescent watched the 5-min movie to establish autonomic baseline data. Next, in the peer session, the peer manipulation was explained to the participant (~13 min); in the solo session participants read magazines for the same time period. Then, participants performed the BART (with or without peer influence, depending on the session). There was a 3-min break after the first BART block of 15 trials. In the peer session, the adolescent interacted with the peer during this break (detailed procedures in Supplementary Materials 2). To control for potential effects of communication in general, the experimenter briefly talked with the adolescent during this 3-min break in the solo session. After the BART, participants completed questionnaires and performed tasks irrelevant to the current study. Parallel to the second and third session with the adolescent, the DISC was administered to one of the parents/caretakers (3–4 h).

The session was postponed if participants had smoked in the last hour before the assessment or used drugs or alcohol in the last 24 h. The second and third session were scheduled at the same time on different days, with a maximum 2-week interval.

### Data-Analysis

A two-tiered data-analytic approach was utilized. In Tier I, analyses of behavioral data were performed as preregistered (Supplementary Materials 1; https://aspredicted.org/gh3u4.pdf). In the *primary analyses*, a 2 (group) by 2 (condition) repeated measures ANOVA was used with age and SES as potential covariates (only if groups differed), with and without intelligence as covariate given the controversy on this topic (Dennis et al., 2009). In the *secondary analyses*, separate repeated measures ANOVA’s were performed to assess the influence of ADHD presentation, medication use, comorbid Disruptive Behavior Disorders (DBD), anxiety-, mood- and substance use disorders, and autism symptoms.

In Tier II, non-preregistered analyses were performed on the physiological data. Paired-samples *t*-tests were performed to check whether HR was higher and PEP and RSA were lower during task conditions relative to baseline (there was a separate baseline measurement in both sessions). To estimate the effect of ADHD on autonomic reactivity, three separate 2 (group) by 2 (condition) repeated measures ANOVA’s were performed with ΔHR, ΔPEP and ΔRSA as outcome variables, respectively.^2^ Independent *t*-tests within the ADHD group were performed to assess the influence of comorbid DBD.

To test whether the difference in autonomic reactivity between conditions was associated with the difference in risk taking between conditions, we conducted three regression analyses (for HR, PEP, RSA, respectively). Therefore, we defined:

1 $$ \mathrm{Behavioral}\ \mathrm{peer}\ \mathrm{index}=\left(\left({\mathrm{BART}}_{\mathrm{peer}}-{\mathrm{BART}}_{\mathrm{solo}}\right)/{\mathrm{BART}}_{\mathrm{solo}}\right)\times 100\% $$

2 $$ \mathrm{Autonomic}\ \mathrm{peer}\ \mathrm{index}=\left(\left(\varDelta {\mathrm{ANS}}_{\mathrm{peer}}-\varDelta {\mathrm{ANS}}_{\mathrm{solo}}\right)/\varDelta {\mathrm{ANS}}_{\mathrm{solo}}\right)\times 100\% $$

For all analyses, outliers were detected based on absolute deviation around the median, using a threshold of 2.5 times the median absolute deviation (Leys et al., 2013).^3^ As preregistered, we tested whether outliers influenced the results by running all analyses with and without outliers. Analyses without outliers are reported, analyses with outliers can be found in Supplementary Materials 3, and when including or excluding outliers influenced the results in terms of significance, this was indicated explicitly. Specific information on outliers and missing data for all analyses are reported in Supplementary Materials 3.

## Results

### Power

The minimum preregistered sample size of 80 participants per group was reached. A power calculation using G*Power (Faul et al., 2007) indicated that given the current sample size, *α* = 0.05, 1 – *β* = 0.80, and an estimated between condition correlation of 0.5, small within group and within × between group interaction effects (*f* = 0.10), and small-to-medium between group effects (*f* = 0.18) could be detected.

### Group Characteristics

Groups did not differ on age, intelligence and SES. The ADHD group displayed higher scores on all DBDRS subscales, higher levels of autistic symptoms and a lower body mass index (BMI) relative to TD adolescents (see Table 1 for demographic characteristics).

### Validity Check of the Peer Influence Manipulation

To assess the success of the peer influence manipulation, screenshots of all WhatsApp conversations between the participants and the confederate were investigated. Screenshots of all WhatsApp conversations were scored by two independent coders and were classified into one of three categories: (1) explicit reaction to peer encouragement (e.g., “thanks for the suggestion”, “no way I’m gonna pump the balloon any further”); (2) short/neutral reaction to the peer encouragement (e.g., “yes”, “okay”) but active participation in the conversation with the peer and (3) distrustful/skeptical remarks indicating not trusting the peer was real. A total of 155 participants explicitly reacted to the peer encouragement, 25 participants only reacted briefly to the peer encouragement but actively participated in the rest of the WhatsApp conversation, and distrustful or skeptical remarks were not coded by any rater. The inter-rater reliability was high (93%) and disagreement between coders was solved by debate. This confirmed that the manipulation was successful in all participants.

### Tier I: Behavioral Analyses

#### Primary, Preregistered Analyses

A 2 (condition) by 2 (group) repeated measures ANOVA revealed an effect of condition (*F*(1,168) = 50.35, *p* < 0.001, *η*_*p*_^*2*^ = 0.23), no effect of group (*F*(1,168) = 0.29, *p* = 0.59, *η*_*p*_^*2*^ = 0.002) and no interaction between condition and group (*F*(1,168) = 2.17, *p* = 0.14, *η*_*p*_^*2*^ = 0.01). That is, decision making was more risky in the peer condition than in the solo condition.^4^ However, adolescents with ADHD did not differ in risk taking from controls, and both groups demonstrated similar susceptibility to peer influence (Fig. 1).

#### Secondary, Pre-Registered Analyses

Secondary preregistered analyses testing effects of ADHD presentations, medication, comorbid DBD, comorbid anxiety disorders or autism symptoms all revealed the same pattern as the primary analyses. Moreover, adding intelligence as a covariate or leaving outliers into the sample did not affect the results. Findings are described in more detail in Supplementary Materials 3.

### Tier II: Autonomic Reactivity

#### Manipulation Check

An increase in HR and decrease in PEP and RSA from baseline to task was observed in both conditions (see Supplementary Materials 3 for analyses), which suggests that physiological stress was higher during task execution than during baseline.

#### Autonomic Reactivity to Peer Influence

A 2 (condition) by 2 (group) repeated measures ANOVA on ΔHR revealed a significant effect of condition (*F*(1,160) = 17.08, *p* < 0.001, *η*_*p*_^*2*^ = 0.10), no effect of group (*F*(1,160) = 0.23, *p* = 0.64, *η*_*p*_^*2*^ = 0.001) and no group-by-condition interaction (*F*(1,160) = 0.05, *p* = 0.82, *η*_*p*_^*2*^ = 0.00), indicating that HR increased more in the peer than the solo condition (Table 2). Results were highly similar when outliers were not removed.

**Table 2:** Means and standard deviations (in parentheses) on the percentage of change in autonomic activity (indexed by ΔHR, ΔRSA and ΔPEP), for adolescents with ADHD and typically developing (TD) adolescents, separated for the solo and peer condition

	ADHD		TD	
	Solo	Peer	Solo	Peer
ΔHR	2.00 (7.09)	4.64 (9.50)	1.58 (7.34)	3.95 (9.01)
ΔRSA	−10.46 (27.41)	−9.64 (25.81)	−4.32 (26.97)	−9.02 (26.21)
ΔPEP	−2.04 (3.58)	−5.07 (3.97)	−3.58 (4.01)	−5.80 (4.43)

The same ANOVA on ΔRSA revealed no effect of condition (*F*(1,146) = 0.63, *p* = 0.43, *η*_*p*_^*2*^ = 0.004), no effect of group (*F*(1,146) = 0.84, *p* = 0.36, *η*_*p*_^*2*^ = 0.01) and no group-by-condition interaction (*F*(1,146) = 1.27, *p* = 0.26, *η*_*p*_^*2*^ = 0.01). Results were highly similar when outliers were not removed.

The same ANOVA on ΔPEP revealed a significant effect of condition (*F*(1,148) = 39.06, *p* < 0.001, *η*_*p*_^*2*^ = 0.21), a significant effect of group (*F*(1,148) = 4.81, *p* = 0.03, *η*_*p*_^*2*^ = 0.03), but no group-by-condition interaction (*F*(1,148) = 0.92, *p* = 0.34, *η*_*p*_^*2*^ = 0.01).^5^ As can be seen in Table 2, the condition effect indicates there is a larger increase in sympathetic activity in the peer relative to the solo condition. The effect of group indicates that TD adolescents, relative to adolescents with ADHD, showed a larger increase in sympathetic activity from baseline to both task conditions. When outliers were not excluded, the effect of group did not reach significance (*p* = 0.07); all other effects were highly similar.

#### Influence of Comorbid DBD

Within the ADHD group, subgroups with and without comorbid DBD did not differ in autonomic reactivity in both conditions and for all three indices, indicating that ADHD-effects are unlikely to be driven by comorbid DBD.

#### Link Between Autonomic and Behavioral Effects of the Peer Manipulation

**Fig. 2:**
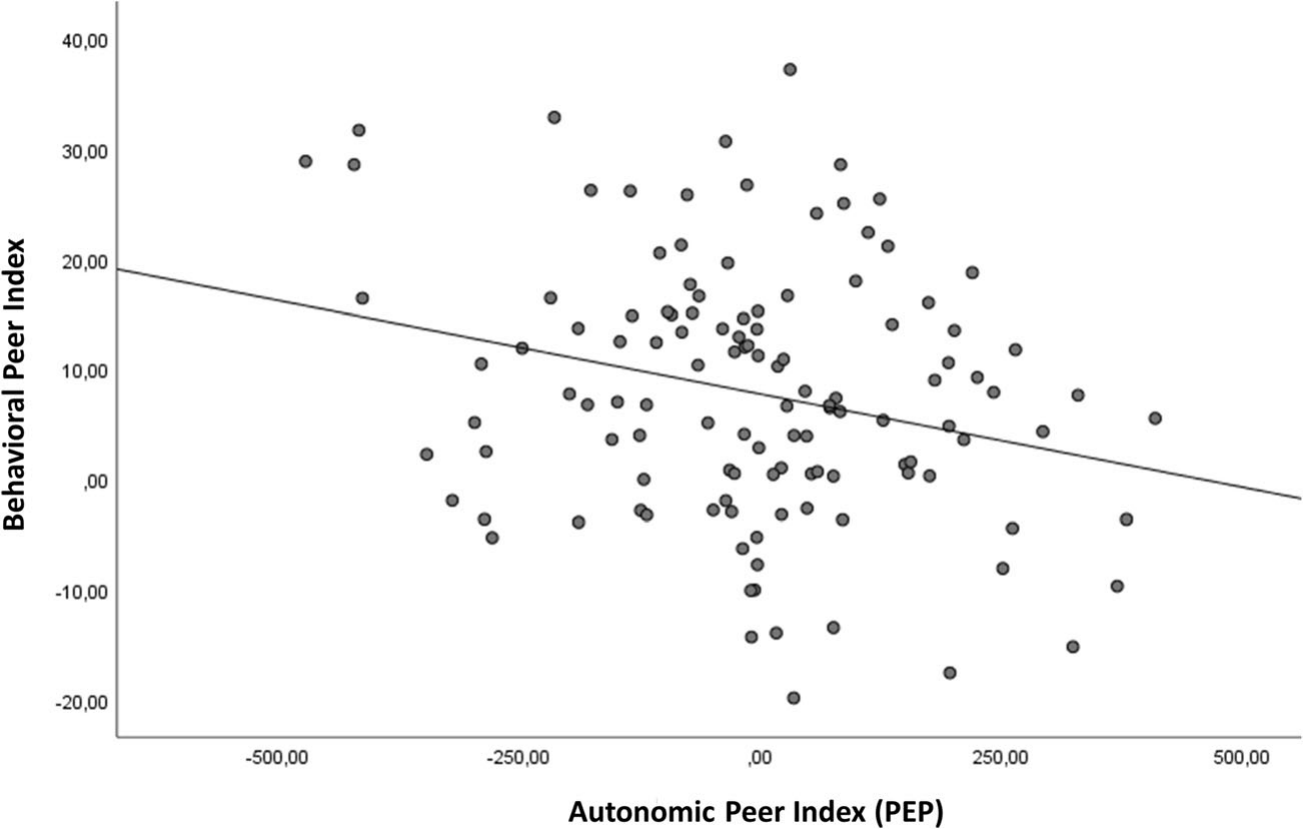
Relationship between the behavioral peer index (Eq. 1; y-axis) and the autonomic peer index (Eq. 2) for PEP (x-axis)

We tested whether the autonomic effect of the peer manipulation was related to its behavioral effect. This was not the case for HR and RSA, *β* = −0.14, *t*(119) = −1.54, *p* = 0.13 and *β* = −0.02, *t*(115) = −0.18, *p* = 0.86, respectively, but was the case for PEP, *β* = −0.25, *t*(120) = −2.86, *p* = 0.005. This indicates that increased sympathetic reactivity to peer influence is related to a larger behavioral susceptibility to peer influence (Fig. 2).

## Discussion

### Summary of Findings

Susceptibility to peer influence increases risk-taking behavior (RTB) in adolescence. In the current study we investigated whether this is particularly the case for adolescents with ADHD. We reasoned that increased susceptibility is likely, because of an ADHD-related enlarged imbalance between socio-emotional and control brain systems and because adolescents with ADHD encounter a wide range of social problems. The increased susceptibility prediction was not supported by the current data. The key finding was that *all* adolescents engaged in higher levels of risk taking under peer influence relative to performing the task alone, regardless of ADHD status. This effect was robust: Follow-up analyses found no differences when comorbid DBD, anxiety disorders or autism symptoms were disentangled, results were similar for all ADHD presentations, were not affected by participants’ medication status, and adding intelligence as potential covariate did not influence the pattern of results. These results therefore imply that the previously observed larger imbalance between socioemotional and cognitive control systems in adolescents with ADHD as well as the well-documented social problems they encounter, do not lead to an increased susceptibility to peer influence over and beyond the susceptibility to peer influence that is generally observed in adolescence (Steinberg, 2007).

#### Autonomic Effects

Two physiological findings warrant further discussion. First, between-group differences were only observed on autonomic reactivity when indexed by PEP, and not when indexed by HR or RSA. Adolescents with ADHD were characterized by less sympathetic reactivity (as indicated by ΔPEP) than TD adolescents. This pattern of autonomic hypoactivation in ADHD is in line with a recent meta-analysis, although most studies on the effect between ADHD and ANS functioning investigate this during resting state or cognitive tasks (Bellato et al., 2020). Therefore, the current study adds the social component to previous studies investigating autonomic differences between adolescents with and without ADHD.

Second, the increase in risk taking as a consequence of peer influence (i.e., the behavioral peer effect) was associated with the change in PEP: adolescents who experience peer influence as stressful (indicated by larger ΔPEP in the peer condition than the solo condition) engage in more risk taking under peer influence. An explanation for this finding could be that those adolescents who experience low physiological stress from peer influence were better able to resist or ignore the risk-encouraging advices from the peer, whereas those experiencing the peer encouragement towards risk taking as stressful – potentially caused by fear of exclusion and a stronger sense of need-to-belong (Baumeister et al., 2005; Pickett et al., 2004) – are more inclined towards adhering to the risk-encouraging advices of the peer. Future studies, however, are needed to further test this explanation, for example by directly measuring fear of exclusion.

Autonomic reactivity only predicted peer-induced risk taking when indexed by PEP, and not when indexed by HR or RSA. An explanation for this finding could be that the acute stress response is predominantly regulated by the sympathetic nervous system (indexed by PEP; see Chrousos, 2009), whereas the parasympathetic nervous system (indexed by RSA) is more often associated with trait-like indices of emotion regulation capabilities (Beauchaine et al., 2013; Beauchaine & Thayer, 2015; Thayer et al., 2012). The pattern of results suggests that specifically the sympathetically-driven stress response is predictive of risk taking.

#### Strengths and Limitations

The lack of difference in risk taking between groups as a function of ADHD is at odds with a rich body of literature demonstrating increased engagement in real-life RTB in adolescents with ADHD (Nigg, 2013; Pollak et al., 2019). However, differences in performance on gambling tasks are typically small and findings are inconsistent. A comprehensive meta-analysis only found a small-to-medium effect size (*d* = 0.36) for the difference between ADHD and TD groups in risk taking on gambling tasks (Dekkers et al., 2016). Six studies in this meta-analysis used the BART and between-group effect sizes were highly heterogeneous ranging from *d* = −0.23 to *d* = 0.46. Relatively low ecological validity of gambling tasks could explain the discrepancy with real-life findings (Pollak et al., 2018). Notwithstanding the lack of group differences, the addition of peer-influence manipulations could be a useful first step to increase this ecological validity, and future studies should continue to bridge the gap between real-life and experimental risk taking.

Despite several notable strengths (preregistration of participant selection, peer influence manipulation and behavioral data-analytic approach, large sample, rigorous assessment of ADHD and comorbidity), some limitations warrant consideration. First, it might be argued that the current peer influence manipulation was not strong enough. However, we consider this unlikely for three reasons: (i) risk taking clearly increased in the peer influence condition relative to the solo condition; (ii) physiological findings demonstrate a larger baseline-to-task increase in HR and decrease in PEP in the peer condition relative to the solo condition, indicating that the virtual peer increased physiological stress and (iii) evaluation of all screenshots of the conversations with the virtual peer by two coders indicated that the manipulation was trustworthy for all adolescents.

Second, our power calculation was aimed at establishing sufficient power for the primary preregistered analysis, and potentially some of the follow-up subgroup analyses might be underpowered. Moreover, studies linking physiology to behavior often report small effect sizes (Fanti et al., 2019; Portnoy & Farrington, 2015) – which potentially stayed undetected in the current study. Large preregistered studies on the autonomic effects of peer influence are warranted to test the hypothesis derived from our results that especially SNS reactivity to peers affects the behavioral susceptibility to peer influence.

Third, we only included boys in our study. Evidence is mixed for sex differences in susceptibility to peer influence: some studies report higher susceptibility in boys than girls (e.g., Sumter et al., 2009), some lower susceptibility (e.g., Shepherd et al., 2011) and others report no sex differences (e.g., Dekkers et al., 2019; Gardner & Steinberg, 2005). Future research to elucidate potential sex differences in susceptibility to peer influence is needed, especially in relation to ADHD.

Fourth, only parent report was used to establish the ADHD diagnosis. However, all adolescents who participated already had a prior ADHD diagnosis made by a clinician, which strengthens our confidence in the validity of the diagnosis.

#### Clinical Implications

The finding that adolescents with ADHD are equally susceptible to peer influence as adolescents without ADHD does not imply that reducing the impact of peer influence should not be a treatment goal in adolescents with ADHD. On the contrary, adolescents with ADHD more often encounter peer rejection in daily life (de Boo & Prins, 2007; Hoza, 2007) and their social problems increase the probability of getting involved with deviant peers (Bagwell et al., 2001). So although the mechanism towards RTB (susceptibility to peer influence) may be similar for all adolescents regardless of ADHD status, the *likelihood* of getting into a situation in which peer influence towards RTB occurs (i.e., being involved with deviant peers) may be substantially larger in adolescents with ADHD (Capaldi et al., 2001; Marshal & Molina, 2006).

## Electronic supplementary material

ESM 1 (PDF 90 kb)

ESM 2 (DOCX 30 kb)

ESM 3 (DOCX 33 kb)

## Footnotes

1 Adolescents participated in a multi-experiment study. For purposes irrelevant to the current investigation, hormones were measured and therefore only boys were included.

2 Smoking, age, BMI and exercise have all been implicated in baseline ANS functioning (Hu et al., 2017; Koenig et al., 2014). Their influence on ANS reactivity is less clear. We controlled this by adding these variables to all analyses on ANS reactivity, and reported when results changed in terms of significance.

3 Autonomic peer indices were calculated separately for HR, PEP and RSA.

4 This effect did not differ between adolescents that might have had doubts about the peer manipulation and adolescents that obviously believed in the manipulation (i.e., short/neutral reaction to the peer vs. explicit reaction to the peer; see Supplementary Materials 3 for the analysis). Further note that there was no main effect of task order, but there was an interaction effect between condition and task order, indicating that the effect of peer influence was larger when the peer condition was administered in the third relative to the second test session (see Supplementary Materials 3 for analyses). However, the main effect of condition remained after task order was included as additional factor.

5 A significant interaction effect of smoking and task condition was observed (more specifically, smoking correlated positively with ΔPEP in the peer condition and negatively to ΔPEP in the solo condition). However, including smoking in the model did not change any of the effects in terms of significance.
