# Supplementary material for: Risk Taking by Adolescents with Attention-Deficit/Hyperactivity Disorder (ADHD): a Behavioral and Psychophysiological Investigation of Peer Influence
Source: J Abnorm Child Psychol. 2020 Jun 30;48(9):1129–41. doi: 10.1007/s10802-020-00666-z (PMC7392932; doi:10.1007/s10802-020-00666-z)
Supplement: Supplementary file 1 — (PDF 90 kb) [file 10802_2020_666_MOESM1_ESM.pdf]

ASPREDICTED

Dekkers_BART_PeerPressure (#1846)

Author(s)
Tycho Dekkers (University of Amsterdam) - t.j.dekkers@uva.nl
Hilde Huizenga (University of Amsterdam) - h.m.huizenga@uva.nl
Arne Popma (De Bascule/Leiden University) - a.popma@debascule.com
Brenda Jansen (University of Amsterdam) - b.r.j.jansen@uva.nl
Anika Bexkens (Leiden University) - a.bexkens@fsw.leidenuniv.nl

Created: 11/07/2016 01:28 AM (PT)
Public: 09/06/2019 01:13 AM (PT)

1) Have any data been collected for this study already?
No, no data have been collected for this study yet

2) What's the main question being asked or hypothesis being tested in this study?
H1: Male adolescents (12-18 years old) take more risk on a gambling task under conditions of peer pressure than in a solo condition (effect of condition);
H2: Male adolescents (12-18 years old) with ADHD take more risk on a gambling task than male adolescents (12-18 years old) without ADHD (effect of group);
H3: The difference in risk taking between the conditions (cf. H1) is larger for adolescents with ADHD than for adolescents without ADHD (group * condition interaction).

3) Describe the key dependent variable(s) specifying how they will be measured.
Risk taking is measured with the Balloon Analogue Risk Task (BART). We will use a modified version of the BART, which consists of two blocks, each containing 15 balloons. The explosion points are semi-randomly generated with a mean explosion point at 64 pumps, and the same array of explosion points is used for all participants, and for both conditions. The outcome measure is the mean number of pumps on unexploded trials.
The following covariates will also be assessed.
• Socio-economic status will be measured by asking both parents their highest level of education. This is scored on a scale from 1 to 7, based on Verhage (1966).
• Age measured in years, months and days.
• In order to estimate intelligence level, in both ADHD and control participants, a shortened version of the WISC-III or the WAIS-IV (depending on age; 12-16 years WISC/17 years WAIS) will be administered. For the WISC-III, the subtests Vocabulary and Block Design, which show good correlation with the total IQ, will be used (Sattler, 2001; cf. Dovis et al., 2013). For, adolescents older than 16 years, WAIS-IV subtests Vocabulary and Matrix Reasoning will be used (Pierson, Kilmer, Rothlisberg, & McIntosh, 2012; cf. van Rooij et al., 2015, Mostert et al., 2015).
• All participants are required to refrain from stimulant medication at the day of testing. Regular medication use will be assessed by asking the parents/daily caretakers of the adolescents what medication is used normally.

4) How many and which conditions will participants be assigned to?
The BART will be administered twice to every participant. Sessions are on different days, with a maximum interval of two weeks. In both conditions, the BART consists of two practice blocks. Practice block one consists of three items on which the participant practices the pumping of the balloon (i.e. no money can be cashed on these items). In the second practice block, participants practice with cashing as well for 5 items. Then, two experimental blocks of 15 balloons each are administered (for more information, see point 3).
In one condition, participants will perform the BART alone. In the other condition, the BART will be embedded in a virtual peer pressure manipulation. In this condition, the participant will get to know a “peer” (in fact a research confederate) via a short introduction on WhatsApp (participants receive a phone from the researcher), and is monitored by this peer via a camera. The participant is instructed that the peer is involved in another study and that the peer will monitor him during the task and that the peer has to predict the participants’ performance on the task based on a small amount of information (name, age, school, class, hobbies, selfie) that will be derived from the short introduction on WhatsApp. After the practice block before the task, as well as during the break in the task (i.e. after the first 15 trials), the peer will send the participant a risk-encouraging message. A pilot study has confirmed that the manipulation was highly trustworthy. The order of the tasks is counterbalanced: half of the participants starts with the peer session, the other half with the solo session (based on odd-even participant numbers).
Based on judgment of the Whatsapp conversation, it will be determined whether the participant believed the peer pressure manipulation or not (yes/no). This will be determined before analyzing the data.
Group membership (i.e. ADHD: yes/no) is established in multiple ways, in an intake session before administration of the BART. Parents of adolescents in the control group will fill out the Disruptive Behavior Disorder Rating Scale (DBDRS), and adolescents will only be included if their scores on ADHD, ODD and CD will all be in the normal range according to the manual. Adolescents will be included in the ADHD group if the following three conditions are all met: (a) they have been diagnosed with ADHD by a mental healthcare professional; (b) their score on the ADHD scale of the DBDRS will be in the subclinical or clinical range according to the manual and (c) a DSM-IV ADHD diagnosis is established after administration of a semi-structured parent interview, i.e. DISC-IV. Scoring of the DISC-IV will take place using the scoring manual of the DISC-IV. This manual leads to a dichotomous outcome on ADHD diagnosis (yes or no). If it yields an ADHD diagnosis, the subtype is automatically established a

5) Specify exactly which analyses you will conduct to examine the main question/hypothesis.

5.1 A 2 (between: group [ADHD, Control]) * 2 (within: condition [solo, peer pressure]) repeated measures ANOVA will be used with age and socio-economic status as potential covariates (but only if if groups differ significantly on these variables).
5.2 A second analysis will be performed with intelligence also added as covariate, as there is some controversy about this topic (e.g. Dennis et al., 2009).

For both 5.1 and 5.2, the mean between subjects effects as well as the effects on the difference between the conditions (within subjects) are of interest.

For all analyses, we will use SPSS. The alpha level of all analyses is 0.05.

6) Any secondary analyses?
6.1 Potential differences between ADHD subtypes will be analyzed with a 4 (between: subtype [combined, inattentive, hyperactive/impulsive, none (in case of controls)]) * 2 (within: condition) repeated measures ANOVA, with the same potential covariates as in the primary analyses described above. Contrasts will be defined such that all subtypes can be compared with each other, as well as with the control group. Because of multiple comparisons we will use a Bonferroni correction.
6.2 Potential differences between adolescents with and without usually taking stimulant medication will be analyzed with a 3 (between: medication (yes or no in ADHD group; not applicable in control group)) * 2 (within: condition) repeated measures ANOVA, with the same potential covariates as in the primary analyses described above
6.3 Potential differences between adolescents with and without co-morbid disruptive behavioral disorders (DBD; i.e. ODD and/or CD; according to DSM-IV) will be analyzed with a 3 (between: DBD yes/no (in ADHD group) or not applicable in Control group) * 2 (within: condition) repeated measures ANOVA, with the same potential covariates as in the primary analyses described above.
6.4 Potential differences between adolescents with and without co-morbid substance use disorders (SUD; according to DSM-IV) will be analyzed with a 2 (between: SUD yes/no) * 2 (within: condition) repeated measures ANOVA, with the same potential covariates as in the primary analyses described above.
6.5 Potential differences between adolescents with and without co-morbid anxiety disorder(s) (according to DSM-IV) will be analyzed with a 2 (between: anxiety disorder yes/no) * 2 (within: condition) repeated measures ANOVA, with the same potential covariates as in the primary analyses described above.
6.6 Potential differences between adolescents with and without co-morbid mood disorders (according to DSM-IV) will be analyzed with a 2 (between: mood disorder yes/no) * 2 (within: condition) repeated measures ANOVA, with the same potential covariates as in the primary analyses described above.
6.7 Effects of autistic symptoms (based on SEV) will be analyzed with a between (score on autism subscale) * 2 (between: group) * 2 (within: condition) repeated measures ANOVA, with the same potential covariates as in the primary analyses described above. We will model an interaction effect between autistic symptoms and group. I.e. we add a continuous between subjects variable (i.e. score on autism subscale of the SEV) into the repeated measures ANOVA.

7) How many observations will be collected or what will determine sample size? No need to justify decision, but be precise about exactly how the number will be determined.
Based on a-priori power analysis, 123 participants per group (only boys, aged 12-17, IQ >80) are needed. We aim to test 130 participants per group, as we will remove participants with outlying scores. However, data collection will stop in October 2018. If 130 participants per group will not be reached by then, the sample size will be lower (with a minimum of 80 participants per group).

8) Anything else you would like to pre-register? (e.g., data exclusions, variables collected for exploratory purposes, unusual analyses planned?)
• Participants that used stimulant medication at the day of testing will be excluded
• Participants with an estimated IQ below 80 will be excluded
• Participants not meeting criteria for group membership (see above) will be excluded
• Participants who seemed not to believe the peer pressure manipulation based on their reactions in the Whatsapp conversation, will be excluded (see more under point 4)
• Outliers on the BART will be detected based on absolute deviation around the mean, using a threshold of 2.5 times the median absolute deviation (see Leys et al., 2013). We will test whether these outliers influence the results by running all analyses twice: with and without inclusion of the outliers.
