# Supplementary material for: Risk Taking by Adolescents with Attention-Deficit/Hyperactivity Disorder (ADHD): a Behavioral and Psychophysiological Investigation of Peer Influence
Source: J Abnorm Child Psychol. 2020 Jun 30;48(9):1129–41. doi: 10.1007/s10802-020-00666-z (PMC7392932; doi:10.1007/s10802-020-00666-z)
Supplement: Supplementary file 2 — (DOCX 30 kb) [file 10802_2020_666_MOESM2_ESM.docx]

**Supplementary Materials 2: Peer Influence Manipulation**

Two researchers/assistants were involved in the peer influence manipulation. The researcher in the room with the participating adolescent is indicated as the experimenter, the researcher that pretends to be the virtual peer, and who is in another room, is indicated as confederate. Note that the confederate could see and hear the participant at all time on a screen, but the participant never saw the confederate. The protocol below is in chronological order.

1. Start of the session: Experimenter to participant: “Hey, I’m happy you’re on time, because later this session you’ll do something together with another participant in another place”, or (in case of participant being late) “We should hurry a little, because later this session you’ll do something together with another participant in another place”.
2. After rest period: Experimenter installs camera behind participant. Experimenter to participant: “Have a look, the camera is here behind you, so [*name peer*] can watch your screen and can see how you do on the task”.
3. Before explaining BART: Experimenter to participant: “I’ll now explain why the camera is there, and what you have to do next. You’ll do a task on the computer, ‘I’ll explain the task to you in a minute (or, in case the solo session was already administered earlier, “it’s similar to the task with the balloons you did the other day”). The camera is there for another study, that we perform in [*random school in random place in another part of the country*]. Have you ever heard of that school? That school approved to participate in a study, just like you did. The participant over there can watch you through the camera, and in a second he’ll introduce himself to you via WhatsApp. While we were preparing the heart-rate measurement, [*name peer*]^[[1]](#footnote-1)^ already performed the same task you’ll do next. He has to predict your performance based on his own experience, what he sees through the camera and the short introduction via WhatsApp. The goal of that study is to determine whether someone can predict someone else’s performance with very little information. For the introduction you both receive a special phone that belongs to the study. The phone has almost no content; I’ve only put your name in there already [*experimenter has added the name of the participant to the WhatsApp account before the session started*], have a look [*experimenter shows the phone to the participant*]. With this phone you can chat with [*name peer*]. All the rules for the introduction are on this sheet [*experimenter shows instruction sheet*]. You’ll introduce yourself to him quickly, and like shown on this sheet, you are allowed to say your name, age, class, school, and hobbies. Finally, you’ll send him a selfie. You can only say these things to him and nothing else, otherwise [*name peer*] can probably make better predictions than others. [*name peer*] will introduce himself in a second, and then you can introduce yourself as well. I’ll wave to the camera to let them know we’re ready with the instructions. Probably they are already waiting for us. While we were preparing the heart-rate measurement, he was performing the same task you’ll do in a minute.”
4. Instruction sheet for introduction on WhatsApp:

---

INSTRUCTIONS

On another location is another participant that will try to predict your performance on the task you’ll do next, without actually meeting you.

The other participant will introduce himself first. Next, you can introduce yourself by giving him some information about yourself. The things you’re allowed to say are described below.

Don’t tell more than what’s described in these instructions.

Introduce yourself by providing the following information:

- Your name
- Your age
- Your school and grade
- What you like to do in your free time
- Send a selfie

During the break, the other participant will send you another message in which he says how he thinks you’re doing on the task. Feel free to respond shortly.

---

1. Introduction of peer protocol for confederate (acting like peer, in another room), ‘>’ indicates that the confederate has to send a message to the participant.

> Hey

> [personalized message to increase credibility, e.g., “wow, I like your red t-shirt”^[[2]](#footnote-2)^; by the experimenter in the room to the confederate, which occurred out of the participants’ field of view while he was completing questionnaires]

[*wait for response of participant*]

> My name is [*randomized*]

> What’s your name?

[*wait for response of participant*]

> I’m [*age maximally one year apart form age of participant*] years old and I’m in [*number of class maximally one different from participant’s class*] class of [*random name of school in a town far away from where the participant lives*].

> How about you?

[*wait for response of participant*]

> In my free time I often play soccer and I like chilling and gaming

> [*send age-matched selfie from database*]

[*wait for response of participant*]

> can you please ask for the camera to be moved a little bit more to the right/left?

[*experimenter moves the camera as requested by the participant and makes sure the participant asks the virtual peer if it’s good like that*]

> Thanks, it’s perfect now.

> I’ve got to continue now

> Bye

Note that the personalized message, the selfie and the question of moving the camera were all introduced during pilot testing of the protocol, as this proved to increase the credibility of the manipulation. Furthermore, language of the messages was adapted to suit the age-group, and some spelling errors were made on purpose (the same 3 spelling errors were in the protocol for all participants) to enhance credibility.

1. After practice block

Experimenter: “[*name peer*] will try to predict your performance on this task. He will watch you through the camera. Please send him a message that you’re going to start, just to make sure he’s ready.”

Protocol for confederate; again, ‘>’ indicates message by confederate:

[*participant sends message he’s ready*]

> yo, okay

> I would pump the balloons a little more than you just did when you cashed your gains

> good luck with the balloons

*[If participant responds:]*

> I’m not allowed to say anything else, good luck!

1. After 15 of the 30 trials on the BART

Experimenter: “I’ll wave towards the camera to indicate we’re halfway. [*name peer*] has to fill in some questionnaires first, and then he’ll send you a message. In the meantime we can have a short chat [*Experimenter starts short, non-task related chat*].

After 90 seconds, confederate:

> Yo

> Good job

> Although I had thought you would pump them a little further

> For me that worked well

[*wait for response of participant*]

> okay

> I gotta go

1. At the end of the BART.

Confederate:

> Yo

> Well done

> Good luck

> See you later

[*wait for response*]

**Non-scripted situations**

Potential additional reactions of the confederate anytime during experiment:

- If participant makes funny remark: > haha
- If participant makes neutral remark: > okay
- If participant asks a question: > sorry, I’m not allowed to say anything else

1. Names were randomly selected from a list with the 200 most common boys names in the Netherlands in 2017. All participants received messages from a “peer” with a different name. [↑](#footnote-ref-1)
2. Although the confederate was able to see the participant through the camera, the content of the personalized message that should be send was provided by the experimenter to the confederate via text communication (which occurred out of the participants’ field of view). This was done to ensure that this message was highly credible (during the resting period before introducing the peer component, the experimenter had 30 minutes time to come up with a credible, personalized message). Most of the times, the message contained information about either the participants’ clothing or haircut; the message was always positive in tone. [↑](#footnote-ref-2)
