# Supplementary material for: Risk Taking by Adolescents with Attention-Deficit/Hyperactivity Disorder (ADHD): a Behavioral and Psychophysiological Investigation of Peer Influence
Source: J Abnorm Child Psychol. 2020 Jun 30;48(9):1129–41. doi: 10.1007/s10802-020-00666-z (PMC7392932; doi:10.1007/s10802-020-00666-z)
Supplement: Supplementary file 3 — (DOCX 33 kb) [file 10802_2020_666_MOESM3_ESM.docx]

**Supplementary Materials 3: Additional Analyses**

1. **Outlier information Tier I analysis**

***Primary pre-registered analyses***

Ten outliers were detected on the BART in the solo condition (6 ADHD, 4 TD), and three in the peer condition (2 ADHD, 1 TD; these three were also outliers in the solo condition).

1. **Secondary analyses**

***Secondary, pre-registered analyses***

*ADHD Presentation*

A 2 (condition) by 4 (group: ADHD combined presentation, ADHD inattentive presentation, ADHD hyperactive/impulsive presentation, TD) repeated measures ANOVA was conducted to investigate differences between ADHD presentations. Similar to the primary analysis, there was an effect of condition (*F*(1,166) = 4.89, *p* = .03). However, there was no effect of ADHD presentation (*F*(3,166) = .52, *p* = .67) and no interaction between condition and ADHD presentation (*F*(3,166) = 1.00, *p* = .40). Contrasts revealed no significant differences between any of the groups.

*Stimulant medication*

A 2 (condition) by 3 (group: ADHD regularly using medication, ADHD not using medication, TD) repeated measures ANOVA was conducted to assess the effect of medication use. Again, there was an effect of condition (*F*(1,167) = 28.95, *p* < .001, but no effect of medication subgroup (*F*(2,167) = .15, *p* = .86) and no interaction between condition and medication subgroup (*F*(2,167) = 1.36, *p* = .26). Contrasts revealed no significant differences between any of the groups.

*Comorbid DBD*

A 2 (condition) by 3 (group: ADHD with DBD, ADHD without DBD, TD^[[1]](#footnote-1)^) repeated measures ANOVA was conducted to investigate the effect of comorbid DBD. Again, there was an effect of condition (*F*(1,166) = 35.16, *p* < .001), no effect of DBD subgroup (*F* (2,166) = .44, *p* = .65) and no interaction between condition and DBD subgroup (*F*(2,166) = .84, *p* = .44). Contrasts revealed no significant differences between any of the groups.

*Comorbid anxiety disorders*

A 2 (condition) by 3 (group: ADHD with anxiety disorder, ADHD without anxiety disorder, TD) repeated measures ANOVA was conducted to investigate the effect of comorbid anxiety disorders. An effect of condition (*F*(1,166) = 33.92, *p* < .001) was found, but no effect of anxiety disorders subgroup (*F*(2,166) = .23, *p* = .79) and no condition × anxiety disorders subgroup interaction (*F*(2,166) = .84, *p* = .43). Contrasts revealed no significant differences between any of the groups.

*Effects of autistic symptoms*

A 2 (group: ADHD vs TD) by 2 (condition: solo vs peer) repeated measures ANOVA with autistic symptoms as additional between factor revealed an effect of condition (*F*(1,166) = 30.58, *p* < .001), no effect of group (*F*(1,166) = .46, *p* = .42), and no condition × group interaction (*F*(1,166) = .10, *p* = .75). There was no effect of autistic symptoms (*F*(1,166) = 2.85, *p* = .09), no interaction between condition and autistic symptoms (*F*(1,166) = 1.26, *p* = .26) and no interaction between group and autistic symptoms (*F*(1,166) = .51, *p* = .48).

*Substance Use Disorders (SUD) and mood disorders*

Preregistered analyses on comorbid SUD and comorbid mood disorders were not performed as only two and five participants with ADHD met criteria for those disorders, respectively.

As preregistered, all primary and secondary analyses were also performed without removing outliers, with intelligence as additional covariate, and a combination of these two. Results of these analyses are described in Table S1 below.

|  |  | **Without removing outliers** | **With intelligence as covariate** | **Without removing outliers and with intelligence as covariate** |
| --- | --- | --- | --- | --- |
| Primary analysis | Condition | *F*(1,178) = 55.6,  *p* < .001 | *F*(1,167) = 50.0,  *p* < .001 | *F*(1,177) = 55.3,  *p* < .001 |
|  | Group | *F*(1,178) = .003,  *p* = .96 | *F*(1,167) = .2,  *p* = .66 | *F*(1,177) = .04,  *p* = .83 |
|  | Condition × Group | *F*(1,178) = .7,  *p* = .41 | *F*(1,167) = 2.3,  *p* = .13 | *F*(1,177) = .7,  *p* = .42 |
|  | Intelligence | N/A | *F*(1,167) = 1.7,  *p* = .20 | *F*(1,177) = 6.7,  *p* = .01 |
|  | Condition × Intelligence | N/A | *F*(1,167) = .3,  *p* = .57 | *F*(1,177) = .02,  *p* = .88 |
| Secondary analysis: ADHD presentation (combined, inattentive, hyp./imp., TD control) | Condition | *F*(1,176) = 5.4,  *p* = .02 | *F*(1,165) = 5.0,  *p* = .03 | *F*(1,175) = 5.3,  *p* = .02 |
|  | ADHD presentation | *F*(3,176) = .1,  *p* = .98 | *F*(3,165) = .5,  *p* = .71 | *F*(3,175) = .1,  *p* = .97 |
|  | Condition × ADHD presentation | *F*(3,176) = .3,  *p* = .81 | *F*(3,165) = .4,  *p* = .39 | *F*(3,175) = .3,  *p* = .81 |
|  | Intelligence | N/A | *F*(1,165) = 1.6,  *p* = .21 | *F*(1,175) = 6.6,  *p* = .01 |
|  | Condition × Intelligence | N/A | *F*(1,165) = .3,  *p* = .60 | *F*(1,175) = .04,  *p* = .84 |
| Secondary analysis: stimulant medication (yes, no, TD control) | Condition | *F*(1,177) = 31.3,  *p* < .001 | *F*(1,166) = 28.7,  *p* < .001 | *F*(1,176) = 31.1,  *p* < .001 |
|  | Medication subgroup | *F*(2,177) = .2,  *p* = .81 | *F*(2,166) = .1,  *p* = .91 | *F*(2,176) = .3,  *p* = .73 |
|  | Condition × Medication subgroup | *F*(2,177) = 1.1,  *p* = .35 | *F*(2,166) = 1.4,  *p* = .25 | *F*(2,176) = 1.1,  *p* = .35 |
|  | Intelligence | N/A | *F*(1,166) = 1.7,  *p* = .20 | *F*(1,176) = 6.9,  *p* = .01 |
|  | Condition × Intelligence | N/A | *F*(1,166) = .3,  *p* = .60 | *F*(1,176) = .04,  *p* = .84 |
| Secondary analysis: comorbid DBD (yes, no, TD control) | Condition | *F*(1,176) = 41.6,  *p* < .001 | *F*(1,165) = 35.0,  *p* < .001 | *F*(1,175) = 41.3,  *p* < .001 |
|  | DBD subgroup | *F*(2,176) = .004,  *p* = .99 | *F*(2,165) = .3,  *p* = .75 | *F*(2,175) = .03,  *p* = .97 |
|  | Condition × DBD subgroup | *F*(2,176) = .2,  *p* = .81 | *F*(2,165) = .9,  *p* = .42 | *F*(2,175) = .2,  *p* = .82 |
|  | Intelligence | N/A | *F*(1,165) = 1.4,  *p* = .24 | *F*(1,175) = 6.5,  *p* = .01 |
|  | Condition × Intelligence | N/A | *F*(1,165) = .2,  *p* = .66 | *F*(1,175) = .1,  *p* = .79 |
| Secondary analysis: comorbid anxiety disorders (yes, no, TD control) | Condition | *F*(1,176) = 46.1,  *p* < .001 | *F*(1,165) = 33.3,  *p* < .001 | *F*(1,175) = 45.9,  *p* < .001 |
|  | Anx. subgroup | *F*(2,176) = .7,  *p* = .50 | *F*(2,165) = .2,  *p* = .82 | *F*(2,175) = .8,  *p* = .47 |
|  | Condition × Anx. subgroup | *F*(2,176) = .8,  *p* = .44 | *F*(2,165) = .9,  *p* = .42 | *F*(2,175) = .8,  *p* = .45 |
|  | Intelligence | N/A | *F*(1,165) = 1.6,  *p* = .21 | *F*(1,175) = 6.6,  *p* = .01 |
|  | Condition × Intelligence | N/A | *F*(1,165) = .2,  *p* = .66 | *F*(1,175) = .1,  *p* = .78 |
| Secondary analysis: autistic symptoms added as between factor | Condition | *F*(1,176) = 37.1,  *p* < .001 | *F*(1,165) = 29.9,  *p* < .001 | *F*(1,175) = 36.9,  *p* < .001 |
|  | Group | *F*(1,176) = .2,  *p* = .67 | *F*(1,165) = .5,  *p* = .47 | *F*(1,175) = .2,  *p* = .64 |
|  | Condition × Group | *F*(1,176) = 1.0,  *p* = .33 | *F*(1,165) = .1,  *p* = .78 | *F*(1,175) = 1.0,  *p* = .33 |
|  | Autistic symptoms | *F*(1,176) = .8,  *p* = .37 | *F*(1,165) = 2.5,  *p* = .11 | *F*(1,175) = .6,  *p* = .44 |
|  | Autistic Symptoms × Group | *F*(1,176) = 1.0,  *p* = .33 | *F*(1,165) = .5,  *p* = .50 | *F*(1,175) = .7,  *p* = .40 |
|  | Autistic Symptoms × Condition | *F*(1,176) = 1.5,  *p* = .22 | *F*(1,165) = 1.2,  *p* = .28 | *F*(1,175) = 1.6,  *p* = .21 |
|  | Intelligence | N/A | *F*(1,165) = 1.3,  *p* = .25 | *F*(1,175) = 6.3,  *p* = .01 |
|  | Intelligence × Condition | N/A | *F*(1,165) = .2,  *p* = .66 | *F*(1,175) = .03,  *p* = .86 |

**Table S1**. Primary and secondary preregistered analyses with intelligence as additional covariate, without excluding outliers and a combination of these two.

1. **Additional Tier I analyses**

***Reactions to peer manipulation***

The effect of the peer manipulation was not different for participants who reacted explicitly to the peer encouragement (*n*=155) and participants who reacted only shortly and in a neutral way (*n*=25): A 2 (condition) by 2 (coding of screenshots) repeated measures ANOVA revealed an effect of condition (*F*(1,168) = 22.80, *p* < .001), no effect of the coding of the screenshots (*F*(1,168) = .04, *p* = .85) and no interaction between condition and coding of the screenshots (*F*(1,168) = .17, *p* = .68).

***Influence of task order***

The primary analysis was also conducted with task order as additional factor. There was no effect of task order (*F*(1,166) = 3.04, *p* = .08), but there was an interaction between task order and condition (*F*(1,166) = 8.52, *p* < .01), indicating that the effect of condition was stronger for participants who started with the solo session than for those starting with the peer session. However, similar to the primary analysis, the analysis still revealed an effect of condition (*F*(1,166) = 51.80, *p* < .001), no effect of group (*F*(1,166) = .32, *p* = .57) and no interaction between condition and group (*F*(1,166) = .97, *p* = .33). Also, there was no group by task order interaction (*F*(1,166) = .07, *p* = .80).

1. **Manipulation check physiological analyses**

Paired samples *t*-tests were performed to investigate differences in HR, RSA and PEP between baseline and the two BART conditions (a baseline measurement was obtained in both sessions). In both the solo and peer condition, BART relative to baseline, yielded higher HR (*t*(165) = -2.95, *p* = .004, *d* = .23 for solo; *t*(169) = -5.90, *p* < .001, *d* = .45 for peer), lower RSA (*t*(162) = 4.30, *p* < .001, *d* = .34 for solo; *t*(158) = 5.73, *p* < .001, *d* = .45 for peer) and lower PEP (*t*(158) = 9.66, *p* < .001, *d* = .77 for solo; *t*(159) = 16.92, *p* < .001, *d* = 1.33 for peer). Results were highly similar when outliers were not removed. This increase in HR and decrease in PEP and RSA suggests that physiological stress was higher during task execution than during baseline.

1. **Information on outliers and missing data for Tier II analyses on autonomic reactivity to peer influence**

***Missing data***

Data was missing for several participants, either due to technical malfunctioning, irregular respiration or low signal quality. HR data was missing for 7 adolescents in the solo condition (6 ADHD, 1 TD) and 10 adolescents in the peer condition (7 ADHD, 3 TD), RSA data was missing for 12 adolescents in the solo condition (9 ADHD, 3 TD) and 10 adolescents in the peer condition (7 ADHD, 3 TD), and PEP data was missing for 14 adolescents in the solo condition (10 ADHD, 4 TD) and 13 adolescents in the peer condition (10 ADHD, 3 TD).

***Outliers***

Two outliers were detected on ΔHR in the solo condition (both TD), and one in the peer condition (TD). For ΔRSA, five outliers were detected in the solo condition (4 TD, 1 ADHD) and 12 in the peer condition (5 TD, 7 ADHD). For ΔPEP, 7 outliers were detected in the solo condition (5 TD, 2 ADHD) and 8 in the peer condition (4 TD, 4 ADHD). Similar to preregistered analyses, results with and without outliers are reported.

1. **Tier II analyses without excluding outliers**

***Manipulation check***

In both the solo and peer condition, BART relative to baseline, yielded higher HR (*t*(167) = -3.28, *p* =.001 for solo; *t*(170) = -6.01, *p* < .001 for peer), lower RSA (*t*(167) = 3.77, *p* < .001 for solo; *t*(170) = 3.56, *p* < .001 for peer) and lower PEP (*t*(165) = 9.28, *p* < .001 for solo; *t*(167) = 14.36, *p* < .001 for peer).

***Autonomic reactivity to peer influence***

A 2 (condition) by 2 (group) repeated measures ANOVA on ΔHR revealed a significant effect of condition (*F*(1,163) = 16.20, *p* < .001), no effect of group (*F*(1,163) = .001, *p* = .97) and no group-by-condition interaction (*F*(1,163) = .06, *p* = .81), indicating that HR increased more in the peer than the solo condition.

The same ANOVA on ΔRSA revealed no effect of condition (*F*(1,163) = .55, *p* = .46), no effect of group (*F*(1,163) = .69, *p* = .25) and no group-by-condition interaction (*F*(1,163) = 1.35, *p* = .25).

The same ANOVA on ΔPEP revealed a significant effect of condition (*F*(1,159) = 40.00, *p* < .001), no significant effect of group (*F*(1,159) = 3.33, *p* = .07), and no group-by-condition interaction (*F*(1,159) = 3.14, *p* = .08). The condition effect indicates there is a larger increase in sympathetic activity in the peer relative to the solo condition.

***Link between autonomic and behavioral effects of the peer manipulation***

The autonomic effect of the peer manipulation was not related to its behavioral effect, as measured by HR, *β* = -.01, *t*(163) = -.14, *p* = .89, RSA, *β* = .09, *t*(163) = 1.08, *p* = .28, and PEP, *β* = -.14, *t*(163) = -1.77, *p* = .08.

1. Note that for this analysis and for the analysis on comorbid anxiety disorders, data from 1 adolescent with ADHD was missing, as his parents withdrew from DISC participation after one session. [↑](#footnote-ref-1)
